# Editorial: Macrocognition: The Science and Engineering of Sociotechnical Work Systems

**DOI:** 10.3389/fpsyg.2017.00515

**Published:** 2017-04-20

**Authors:** Paul Ward, Robert R. Hoffman, Gareth E. Conway, Jan Maarten Schraagen, David Peebles, Robert J. B. Hutton, Erich J. Petushek

**Affiliations:** ^1^The Applied Cognition & Cognitive Engineering Research Group, University of HuddersfieldHuddersfield, UK; ^2^Florida Institute for Human and Machine CognitionOcala, FL, USA; ^3^Defence Science and Technology Laboratory (Dstl)Porton Down, UK; ^4^TNO Netherlands Organisation for Applied Scientific ResearchSoesterberg, Netherlands; ^5^Trimetis Ltd.Bristol, UK; ^6^College of Human Medicine, Michigan State UniversityEast Lansing, MI, USA

**Keywords:** adaptive thinking, complexity, expertise, human performance, cognition

The increasing complexity of work systems and changes in the nature of workplace technology over the past century have resulted in a substantial shift in the nature of work activities, from those predominated by physical labor toward more cognitively oriented work. Modern work systems have many characteristics that make them cognitively complex: They can be highly interactive; comprised of multiple agents and artifacts; information may be limited, contested, or distributed across space and time; problems can be unexpected and emergent; task goals are frequently ill-defined, conflicting, and dynamic; planning may only be possible at general levels of abstraction or require adaptive solutions; a considerable degree of proficiency or expertise is required; the stakes are often high; and problems usually involve uncertainty, time-constraints, and stress. To complicate matters further, cognition in complex work settings is typically constrained by broader professional, organizational, and institutional practices and policies, which themselves can be a moving target as work systems and organizations adapt to a constantly-changing landscape. These features of cognitive work present significant challenges to scientific methodology and theory, and to subsequent design of reliable work methods and the technologies that shape them.

Historically, philosophers and scientists have used divergent methods to understand the mental activities experienced during cognitive work at multiple levels of analysis. Some have examined cognition at an associative, contextual, functional, or holistic level, relying on naturalistic methods to understand the higher mental processes as they work in harmony during goal-directed behavior. Others have embraced experimental and computational methods and favored internal control over external validity, often reducing cognition to a psychology of fundamental acts, such as short-term memory access and action selection at the millisecond level.

More recently, Macrocognition has evolved as a complementary paradigm, focused on how cognition adapts to complexity, particularly in work settings (Klein et al., 2003). Macrocognitive researchers have studied the cognitive functions and processes associated with skilled, adaptive, collaborative, and resilient cognitive work in the context of the aforementioned complexities of sociotechnical work systems. Typically, this research has been carried out using cognitive task analytic techniques that draw on both naturalistic and experimental methods (e.g., Crandall et al., 2006). The primary goals of research in Macrocognition are to better understand cognitive adaptations to complexity, to increase our theoretical understanding of the organism–environment relations by studying the mapping between cognitive work and real-world demands, to better understand work-as-done rather than work-as-prescribed, work-as-imagined, or work-as-disclosed, and to promote use-inspired research capable of improving system performance and informing theory development (see for instance Schraagen et al., 2008).

The aims of this Research Topic are to showcase some of the exciting research on Macrocognition being conducted by cognitive scientists, cognitive ergonomists, and cognitive systems engineers, and to demonstrate the broad reach of this relatively new discipline. The opening paper, co-authored by one of the pioneers of Naturalistic Decision Making and Macrocognition, Klein and Wright, describes the evolution of this research and identifies some of the key drivers of the origin of Macrocognition. The paper highlights how this discipline has shaped our thinking about core cognitive processes, and our capabilities for developing training, decision support systems, and system design in complex and uncertain environments.

Four papers examine Macrocognition in traditional and non-traditional yet complex work domains. They present research at different levels of analysis using methods ranging from naturalistic techniques and interviews to simulations and experiments. Baber and McMaster demonstrate how UK police forces gather, frame, and share information as a means to coordinate incident response, and manage the associated uncertainties, risk, and resources. Collins et al. examine sports coaches' use of decision-making strategies. Their findings indicate that deliberation is often used as an immediate check on initial intuitions, which are heavily influenced by prior planning and experience level. Brouwers et al. use a novel, simulated rail control task to examine cue utilization. Their data suggest that individuals with greater cue utilization were more effective at routing trains while managing additional sources of cognitive load. Porat et al. report a series of studies that evaluate how many unmanned automata a single operator can supervise and control. They show that experienced operators were able to supervise around 15 systems with a moderate level of automation but can only control up to three effectively. Moreover, teams of operators generally performed better than individuals working alone.

Two papers investigate Macrocognition in team settings and organizational networks. Buchler et al. investigated the assumption that greater information sharing improves situation awareness and organizational effectiveness. Their data suggest that sending many messages can actually decrease the likelihood of attaining shared situation awareness. The similarity between team members in terms of their functions and initial situation awareness levels likely impacted these results, highlighting important issues for networked organizations. Fiore and Wiltshire synthesize a broad set of perspectives on how team cognition occurs in complex collaborative contexts, as well as the artifacts and technology that support team performance. They provide diagnostic guidelines on studying the relationship between artifacts and team cognition and present implications for how to conceptualize team-supporting technology.

Three papers investigate the role of Macrocognition in design. Fadde presents a framework for translating macrocognitive research into the design of instruction to take place in the workplace. He presents a case study that applies macrocognitive training to baseball and highlights the challenges of embedding such training in the work setting. Goode et al. examine how the macrocognitive approach can inform system design, specifically how incident data can be translated into prevention strategies that address the systemic causes of accidents. They argue that the design process needs to be refined to focus design on monitoring and feedback mechanisms that support high-level decisions. Naikar and Elix suggest that to create work systems that are capable of adapting to complexity, all system elements need to be integrated into the design in a way that supports workers' ability to adapt their behavior and the environmental structure in order to handle novelty as well as familiarity. They present an integrated design approach aimed at facilitating system performance through adaptation.

The final paper, by Laurent and Bianchi, offers a critical view of Macrocognition and asks whether it should be distinguished from other forms of cognition. They echo earlier comments by Klein et al. (2003) that Micro- and Macrocognition present research at different levels and scales of analysis. They argue for the development of a multiscale model of cognition, in which context and cognition interact at multiple levels.

These articles demonstrate the diversity of perspectives and methods employed in research on Macrocognition, as well as the pragmatic focus of this research toward leveraging our understanding of how cognition adapts to complexity. We are grateful to all authors for their contributions and hope that this volume provides important insights into Macrocognition research, and a useful resource for research and application in this discipline. We are confident that Macrocognition has staying power, if only because of its complementarity to the traditional micro-cognitive paradigm.

## Author contributions

All authors listed, have made substantial, direct, and intellectual contribution to the work, and approved it for publication.

### Conflict of interest statement

The author RJBH is affiliated with Trimetis Ltd. GC works for the Ministry of Defence (MOD). All views expressed in this article are those of the author and are not made in any officially capacity as a civil servant in the MOD. The other authors declare that the research was conducted in the absence of any commercial or financial relationships that could be construed as a potential conflict of interest.
